# A descriptive study of treatment provision for problem alcohol drinking in adult males in Khayelitsha, Cape Town, South Africa

**DOI:** 10.1186/s12913-017-2643-z

**Published:** 2017-12-04

**Authors:** Amina Saban, Neo Morojele, Leslie London

**Affiliations:** 1 School of Public Health and Family Medicine, Faculty of Health Sciences, University of Cape Town, Cape Town, South Africa; 2 Alcohol, Tobacco and Other Drug Research Unit, South African Medical Research Council, Pretoria, South Africa

**Keywords:** Treatment provision, Alcohol, Problem drinking, Khayelitsha, Cape Town

## Abstract

**Background:**

Poor, Black African males are underrepresented as patients in facilities that treat problem drinking in Cape Town, South Africa. Reasons for this remain unclear, but factors such as the kinds of treatment provided, perceptions of treatment efficacy, social stigma and traditional treatment beliefs have been suggested as possible barriers to treatment seeking. This descriptive study examined the availability and nature of problem drinking treatment facilities in Khayelitsha, a largely poor township of Black, Xhosa-speaking Africans, on the outskirts of Cape Town.

**Methods:**

Seven treatment facilities for problem drinking in adult males were identified using data from the Department of Social Development in the City of Cape Town. Staff members were identified as key informants at each of the treatment facilities, and were interviewed using a structured questionnaire. Twelve interviews were conducted.

**Results:**

Findings indicated that the available alcohol treatment facilities were relatively new, that treatment modalities varied both across and within treatment facilities, and that treatment was provided largely by social workers. Treatment facilities did not accommodate overnight stay for patients, operated during weekday office hours, and commonly referred patients to the same psychiatric hospital.

**Discussion:**

The study provides a baseline for assessing barriers to treatment for problem drinking in Khayelitsha by highlighting the nature of available facilities as playing a predominantly screening role with associated social work services, and a point of referral for admission to a psychiatric institution for treatment. The social and financial implications of such referral are pertinent to the discussion of treatment barriers.

**Conclusions:**

Recommendations are made to inform policy towards locally-provided integrated care to improve treatment provision and access.

## Background

Alcohol use is a major contributor to the global burden of disease, with a verified causal role in wholly alcohol-attributable disease conditions, chronic infectious diseases and acute conditions with alcohol a component cause [1]. Harmful alcohol use (together with tobacco use, an unhealthy diet, and a lack of physical exercise) has been singled out as a major predisposing risk factor for the current high prevalence of non-communicable diseases globally [2]. According to the Global Burden of Disease and Injury 2010 study [3], alcohol use accounted for 2.7 million deaths and 3.9% of global disability adjusted life years lost, with alcohol use accounting for 5.4% of the disease burden amongst men and 2.0% in women. Furthermore, the common occurrence and the increased risk of psychiatric disorder associated with substance use has been well-documented in studies conducted in different geographical regions globally [4].

In South Africa, alcohol consumption and substance use disorders are more prevalent in males [5, 6]. Studies in South Africa also show that the use of alcohol is associated with an increased risk of psychiatric disorder [7] and with huge economic, social and health costs [8]. For example, alcohol abuse has been identified as playing a role in 70% of violent crimes in Cape Town, particularly in the poorer areas of the Cape Flats that lie predominantly on the outskirts of the city. Further, foetal alcohol syndrome rates in the Western Cape Province are reportedly the highest in the world, with estimates of 40.5 to 46.4 per 1000, and 65.2 to 74.2 per 1000 [9]. Alcohol has also been identified as the most common primary substance of use for which treatment is sought in South Africa [10]. However, Myers et al. [11] highlight the underrepresentation of poor Black/African people as patients at substance use treatment facilities, in spite of the general increased demand for treatment [10] and the recognised high levels of substance use in this population group [10].

In this study, we identify and describe treatment facilities for problem drinking adult males in Khayelitsha, a township situated on the outskirts of Cape Town, about 25 km from the Cape Town Central Business District. The area comprises predominantly Xhosa-speaking and poor Black African residents, and is amongst the top 10 precincts for contact crimes, sexual crimes, assault, robbery, carjacking, and kidnapping [12]. Observations from ComaCare/Heads Up!, a non-profit organisation that manages head-injured patients admitted for treatment at Groote Schuur Hospital, a tertiary State hospital in Cape Town, have indicated that their patients are largely male Khayelitsha residents. Individuals who are referred to ComaCare for treatment and management, frequently have suffered injuries in alcohol-related accidents or incidents of violence (personal communication, Ms. Jan Webster, September 2012). The high prevalence of these incidents and injuries might be a consequence of untreated problem drinking amongst Khayelitsha male residents. However, information is lacking about facilities that operate in Khayelitsha to assist with or treat problem drinking. For example, though the Western Cape Resource and Services Directory for the Reduction of Harmful Drug and Alcohol Use [13] lists substance use treatment facilities as part of the services provided by the Department of Social Development Western Cape Substance Abuse Unit, the specific details regarding the services provided by these facilities remain indistinct. Furthermore, although the City of Cape Town municipality, through various oral forums, announced its intention to embark on a programme which will incorporate outpatient substance use treatment at community health facilities, the status of these services remains unclear. It is also suspected that organisations that follow an Alcoholics Anonymous (AA) treatment modality and that operate regularly in the urban areas of Cape Town, do not have equivalent branches in Khayelitsha (personal communication, Ms. Jan Webster, September 2012). These observations have not been critically examined or researched empirically and, therefore, remain speculative.

Factors such as social stigma associated with substance use disorders and negative perceptions about the quality and effectiveness of available treatment might discourage treatment seeking [10]. These factors may also act as barriers to treatment seeking and might, therefore, play a role in the underrepresentation of poor and Black patients at substance use treatment centres [9].

This study forms part of a series of planned research projects that aim to examine the prevalence of problem drinking amongst adult males in Khayelitsha, and to identify factors, including social stigma and cultural factors, that potentially pose barriers to treatment seeking for problem drinking in this group. In this study, we aim to identify and describe treatment facilities for problem drinking adult males in Khayelitsha, with a view to establishing the nature of services available for problem drinking adult males in Kahyelitsha.

## Methods

### Sampling

The units of sampling for this study were treatment centres in Khayelitsha that provide services for the treatment of problem drinking in males. Two main sources were used to identify treatment centres to ensure no available services were missed: a) lists of services registered with the Department of Social Development (DSD) and made available to public and academic institutions, and b) information from the South African Community Epidemiology Network for Drug Use Survey, which provides bi-annual statistical reports on substance use treatment facilities in the Western Cape Province (and other provinces) and the individuals who present for treatment at these facilities. These lists were supplemented by information obtained from key informants about other venues where problem drinking was addressed in Khayelitsha. All DSD-listed treatment centres in Khayelitsha (*n* = 7) were selected for investigation. One additional non-governmental organisation (NGO), which was purported to address problems associated with substance use, had to be excluded because its location was uncertain and its chairperson remained unavailable for an interview for the duration of the study.

### Instruments

An interview schedule/questionnaire was drafted specifically for the purposes of this study. This instrument recorded the name of the treatment centre, its physical address, operating hours (if known), telephone numbers, email address (if provided) and the name and contact details of the listed contact person (if known), as well as the names of various identified key informants and stakeholders, and their contact details, demographic information (including age, gender, academic qualification and job description). The balance of this instrument documented details relating to the nature of the services provided by the treatment centre (including when the problem drinking service was initiated, whether residential and/or outpatient services were provided, bed space, opportunity for overnight stay, costs, duration of treatment provided, staff complement, staff roles, staff qualifications, target patients, appointments/walk-in service, predominating language use, kinds of treatment provided). The questions were largely close-ended, were designed to elicit the information deemed pertinent to the study, and provided the opportunity for explanations and further details to be provided.

### Procedures

Data collection commenced once approval had been received from the University of Cape Town Human Research Ethics Committee (HREC REF 537/2015), the City of Cape Town and the governance bodies of the individual treatment facilities (which was either the director, founder, senior social worker or equivalent at the various facilities). The Khayelitsha Development Forum was informed in writing about the plans to conduct the study.

Each treatment facility was visited in person, and ahead of the commencement of the fieldwork, by the principal investigator and an administrative representative of the DSD, and a DSD social worker who was familiar with both the Khayelitsha area geographically, as well as with the staff at each of the alcohol treatment facilities in the area. This visit served to introduce both the study and the research staff to the treatment facilities, thus facilitating the subsequent arrangement of interviews by a qualified social worker fieldworker who was familiar with both the Khayelitsha area and the arena of substance use, abuse, dependence, management and intervention. Healthcare workers who work in the area as part of the University of Cape Town structures and DSD, as well as members of the Khayelitsha Development Forum assisted the fieldworker to develop familiarity with the area and selected research sites (treatment facilities).

The fieldworker telephonically arranged interviews with each contact person listed on the DSD list for each treatment centre. On meeting each interviewee, the fieldworker informed him/her of the details of the study and requested signed informed consent. One of the questions on the questionnaire included soliciting the contact details of further stakeholders and key informants at/for the treatment centre, as some treatment facilities employed separate staff members for administrative duties, counselling services and community visits. The reason for conducting these additional interviews with further stakeholders at any one facility was to ensure that information about the treatment facility would be covered as comprehensively as possible. Where indicated, each of the latter persons was contacted, an appointment to meet was arranged, and the individual was interviewed at a later date.

The interviews were conducted during working hours in a secure and private environment at the treatment centres and recorded on pen-and-paper questionnaires. An attempt was made to complete the interviews as speedily as possible so as not to burden the interviewee during his/her working hours too much. As such, the interviews were designed to be completed within 45 to 60 min.

All attempts were made to interview all identified key informants within the timeline of the study data collection process. However, two programme managers remained unavailable throughout the data collection process. An alternative key informant was interviewed at one of these facilities. Further investigation for the other facility revealed that the service was provided by an NGO on an ad hoc basis from within the staff room of a school building, but no interview could be arranged for the purposes of the study. The data collection was concluded once the list of key informants had been saturated and the interviews completed for each of the identified treatment centres.

### Data analyses

Responses to the questionnaire that constituted count data were tallied to provide frequencies that could be compared (for example, the number of institutions that were in buildings compared with the number housed in shipping containers). Other data were analysed to describe the treatment centres in terms of the selected items (including, when the problem drinking service was initiated, whether residential and/or outpatient services were provided, bed space, opportunity for overnight stay, costs, duration of treatment provided, staff complement, staff roles, staff qualifications, target patients, appointments/walk-in service, predominant language use, kinds of treatment provided). Where appropriate, these descriptions were tallied and the numbers were compared (for example, how many treatment facilities provided support for the families of problem drinkers compared with how many facilities provided support for only the problem drinkers). Information from additional stakeholders at any one treatment facility was used to augment the data obtained from the identified primary stakeholder at the facility.

## Results

Interviews were conducted with 12 key informants at the seven treatment facilities, with three interviews conducted at one facility and two interviews each at three of the facilities. At each of the remaining three facilities one interview was conducted. Data from additional stakeholders at any one treatment facility did not significantly alter the overall findings obtained from the primary stakeholders.

The ages of key informants who completed the study interview ranged from 28 years to 88 years, with most being aged in their 30s or 40s. Seven key informants were female and five were male. They had occupied their positions for a period of one to 6 years, with job descriptions that included being a clinical social worker, a non-clinical social worker, auxiliary social worker, administrator, public relations officer, marketing manager, and clerk. Most of the key informants performed multiple roles within their organizations. The main treatment communication language was Xhosa at all the facilities, with English and Afrikaans being used as and when needed at all the facilities.

All the treatment facilities were located in or close to residential complexes, main roads in Khayelitsha, and close to major transport hubs. Four treatment facilities were located in structures that were formally built with bricks and mortar. One of these facilities was attached to a Community Health Centre, one facility was located inside a multi-purpose community centre that provided space for sports activities, community craft projects, dance and exercise classes and a gymnasium. One formally built treatment facility occupied space inside a modern office block cum business centre, and one formally built treatment facility was situated inside an older building which provided office space for various businesses. Two identified treatment facilities offered their services from shipping containers that had been placed end-to-end, and re-purposed as tandem offices, consulting rooms and reception areas. One treatment facility structure consisted of a shipping container attached to a bricks and mortar building.

Six of the seven treatment facilities were confirmed as being registered with the DSD. The one treatment facility for which registration was in doubt, was listed as an unregistered facility by its chairperson, and as registered by its administrative office/marketing manager in each of the separate interviews. Staff at the various DSD-listed services appeared to be familiar with staff at other registered services, and were often aware of community services that were provided but for which registration was doubtful. Many staff members were aware that their clients/patients often attended more than one treatment facility.

The majority of treatment facilities had been operating for a period of one to 5 years, while one facility was comparatively longstanding, having been in operation for 18 years. In some places, contradictory details were provided by board members and staff on institutional length of service.

All the treatment facilities selected for the study purported to treat or address problem drinking services. One treatment facility provided services for only alcohol-related problems, while the other facilities provided services for problems associated with multiple substances of abuse. Other than alcohol, the most common substances used by the facilities’ clients were reported to be crystal methamphetamine, cannabis and Mandrax. Cocaine and heroin abuse were rarely encountered across all the treatment facilities, but Wonga, and glue abuse were reported at one treatment facility and Wonga and Nyaope abuse were reported at one other facility. (Wonga/Whunga/Woonga and Nyaope are interchangeable terms for street drugs that consist of blends of various commonly available substances such as cannabis and crystal methamphetamine, or cannabis and heroin, and are believed to contain anti-retroviral medication that is used in the treatment of HIV and AIDS. However, the composition of the drugs is uncertain as it is known to vary. Wonga and Nyaope are particularly common in the poorer areas of South Africa).

Five treatment facilities provided counselling support for substance users as well as for their family members. One treatment facility addressed substance use in only adult males, while all the other treatment facilities provided services for males and females of all ages. All treatment facilities reported having clients that were aged 12 and older, but, other than the one treatment facility that treated only adult males, all the facilities indicated that younger clients would not be turned away. All the treatment facilities reported having a preponderance of male patients/clients.

None of the treatment facilities provided overnight or inpatient services, and detoxification was not provided by any of the treatment facilities surveyed for this study. All services were outpatient services, usually for ambulatory clients. Follow up appointments were scheduled once patients had made contact with the treatment facilities.

Key informants indicated that their facilities commonly referred patients to a psychiatric hospital for detoxification. One treatment facility was found to refer all its patients to a second treatment facility in Khayelitsha. The latter facility would provide a further screen and assessment of the patients and the patients would thereafter be referred to the psychiatric hospital for detoxification and treatment as needed. One Khayelitsha facility reported that it referred its patients to NGOs in the Khayelitsha area for treatment, but did not specify the NGOs to which they referred their patients.

The treatment and management models offered varied both across facilities, and within facilities, with more than one treatment modality being offered at four of the treatment facilities. One facility offered no treatment, management or intervention (only screening and assessment), and two facilities offered only one type of service. The treatment modalities included the Matrix Model (an integrative, time-limited treatment, drawing on different therapeutic approaches that is offered on outpatient basis, and follows a highly-structured program) [14], medical models, spiritual guidance and education, general education regarding substance use, brief motivational interviewing, and models of behavior change. In general, the average duration of treatment programmes offered to each patient was at least 4 weeks. Some treatment facilities reported providing continuous/ongoing support and assistance beyond the four-week period, indefinitely, as needed, or as frequently as the patients chose to access them.

Treatment for alcohol-related problems was generally provided free of charge at all the facilities. However, all facilities welcomed donations towards their general maintenance, while one treatment facility accepted nominal amounts for different treatment options including group therapy, individual therapy sessions, drug testing, and assessment respectively.

The availability of group meetings or branch meetings (such as those offered by Narcotics Anonymous (NA) and AA in other Cape Town suburbs) varied across facilities. Three Khayelitsha facilities clearly offered no additional group meetings, while ad hoc AA and NA sessions were offered at two facilities, but these sessions were largely infrequent. One Khayelitsha treatment facility offered several options to supplement their services. These alternative and supplementary management options included exercises in a gym, motivational talks, and other general support with community visits and counselling. The remaining service provided only branch meetings, and on a sessional basis within the building of another treatment facility.

All the surveyed treatment facilities provided their services in the form of contact from at least two service providers. Service providers most often included a social work therapist, an auxiliary social worker, and/or a community volunteer. Other services were provided by fellow-group members in the two facilities where group work was the main mode of service provision, or by group facilitators in the one facility where focus group discussions were commonly employed. Counsellors and pastors provided services in facilities where spiritual assistance predominated.

One facility employed no additional staff other than the treatment service providers. One treatment facility had a receptionist as an additional staff member, while the remaining five facilities variously reported additional staff including a receptionist and/or an events planner, community volunteers, a secretary, clerk, programme manager, senior managerial or administrative staff in the form of a director/chief executive officer/programme manager (with a personal assistant), as well as fieldworkers and follow-up community workers.

## Discussion

In summary, the results obtained illustrate that a variety of services for substance use-related problems operate essentially free of charge within the Khayelitsha area. Access to these services has been facilitated by having the services located largely within residential communities and close to major transport services. The large majority of treatment facilities had been established relatively recently (one to 5 years), provide professional expertise from mainly social workers, with follow-up and outreach assistance from community workers, and were found to treat problems associated with both substance use in general, and problem drinking. Interaction with staff at the various treatment facilities indicated a healthy camaraderie between staff at different facilities, with staff members having regular interaction regarding the referrals of patients, and a familiarity with individual patients and cases. This is possibly indicative of patients attending a variety of treatment facilities in the Khayelitsha area, as well as an informal collaboration between services provided at the various facilities. Several findings in this study might have implications for adult males in Khayelitsha who seek treatment for problem drinking.

One key finding was that none of the surveyed treatment centres provided facilities for the detoxification of individuals who needed such intervention. Further, the treatment centres had no facilities for inpatient care and essentially screened and assessed patients for problem drinking, referring problem drinkers in need of further intervention to a psychiatric hospital outside the area for treatment, and providing therapy from social workers and support from community or auxilliary social workers for those who are affected by problem drinkers and problem drinking. Thus, though referral to a psychiatric hospital would potentially provide the recommended integrated care for psychiatric comorbidity with the problem drinking [15] in the event of such comorbidity, the burden of additional transport costs to inpatient facilities could discourage treatment seeking at the local facilities [16], considering that the majority of Khayelitsha adult male problem drinkers are likely to be breadwinners or indigent.

This study also found that treatment in the Khayelitsha area largely lacked branch meetings from organisations such as AA and NA that are common in the Cape Town urban areas. Thus, similarly, attending branch meetings in more urban areas outside of Khayelitsha for both the problem drinker and his assisting family may be financially onerous and time-consuming.

Furthermore, the local treatment facilities operated largely during office hours and not on weekends. The financial burden from work days lost may therefore serve as a deterrent to treatment seeking in employed males, especially if the nature of their employment is likely to be piece-work, informal or temporary (and would thus be prioritised above treatment seeking), while the availability of these treatment services only during office hours and only on week days essentially means that individuals in fulltime employment cannot access the available treatment in Khayelitsha.

However, though the economic burden of referral to a psychiatric institution for problem drinking and alcohol-related problems that require intervention beyond social work services might be prohibitive and discourage treatment seeking, the stigma of problem drinking and required treatment might be an additional and significant treatment seeking barrier. For example, Schomerus et al. [17] found that alcohol dependence was less likely to be perceived to be a mental disorder than any other substance-related disorder. The latter research also indicated that individuals with alcohol-related disorders were more likely to be viewed unsympathetically, were held personally responsible for their plight, were likely to be rejected socially and were likely to be discriminated against, and suggested that such stigma might vary in different cultural settings. In addition, the literature indicates that substance use treatment is more likely to be sought when a comorbid psychiatric disorder occurs [18]. Thus, there might be a decreased likelihood of treatment seeking for problem drinking alone amongst Khayelitsha males because of social or culturally-associated stigma and/or comorbid psychiatric disorder being under-diagnosed.

Delay in intervention might also play a role in the underrepresentation of Khayelitsha males at substance use treatment facilities in Cape Town. Johnson et al. [19] found that if the period between initial screening and subsequent follow up exceeds 2 days, there is an increased likelihood of individuals not seeking further intervention. Thus, with the local Khayelitsha treatment facilities providing limited intervention and referring patients to a psychiatric institution, the delay in follow up treatment might deter further treatment seeking.

The study also found that Khayelitsha alcohol treatment facilities have mostly been in operation for a short period. Thus, there might be several males in the area who have not had convenient access to any form of locally-provided assistance with their problem drinking for many years, and have abandoned hope of being able to be assisted. In particular, with the lack of available comprehensive substance use treatment services in the Khayelitsha area for many years, the area might be home to several older men who need assistance with problem drinking but who have their problem drinking, and associated treatment seeking ability, either exacerbated by age-related physical health issues, socioeconomic circumstances, or in keeping with the research findings of the National Comorbidity Survey Replication [20] that individuals older than 65 years are more likely to have a decreased perceived need to seek treatment.

### Limitations

Limitations in the study design might have influenced the results obtained and the discussion of the study findings. For example, though this study was limited to describing the treatment available at local facilities in Khayelitsha, it is unclear if there were other treatment facilities that had not been contacted, either because they are unregistered with any of the local City of Cape Town authorities, or because key informants were unaware of their existence. Although it is a recognised requirement for NGOs to be registered with DSD, staff at the surveyed treatment facilities made clear reference to the possibility of unregistered NGOs that could be functioning in the area; inadequate staff communication and State oversight could also result in confusion (at both staff and community level) about a facility’s registration status, as encountered at the one treatment facility where the chairperson and staff member provided differing and conflicting responses regarding the registration status of the facility.

In addition, this study did not examine the nature of treatment offered at the psychiatric hospital to which patients were referred, and thus no comment can be provided on this option. Also, the study did not explore the nature of the assistance provided by community workers. Therefore, the value of the community service cannot be commented upon.

Furthermore, this study relied on self-reports of staff members at available treatment facilities. Though the reliability of some data was examined with additional key informants in some facilities, not all facilities had multiple key informants who could be interviewed, which might have compromised the findings.

Lastly, even though several treatment facilities for the treatment of problem drinking in adult males in Khayelitsha were located and surveyed, this study did not explore the extent to which potential patients and their families in the community were aware of their existence, location and the services they provide. Community awareness of treatment services will be assessed in subsequent proposed studies, together with an investigation of the demand for problem drinking treatment in the Khayelitsha area.

However, a major strength of this study has been that it has addressed a recognised local knowledge deficit by identifying and describing the availability and nature of treatment facilities in the Khayelitsha area that address problem drinking in adult males. In so doing, the study has been able to provide content about the nature of available services, and identified potential knowledge gaps that might be filled by ongoing research. For example, further investigation could explore i) the role of staff capacity and resources in treatment provision, ii) how facilities could be improved to improve access to treatment locally, and possibly defer referral to a psychiatric hospital, iii) how details about the service provided can be communicated to the community both to improve knowledge about the services and to demystify the treatment, iv) the referral process of the available facilities, v) the reasons for the limited number of branch meetings such as AA and NA, vi) how currently unregistered and potentially illegitimate treatment facilities can be identified, assessed and harnessed to improve community care by altering community perceptions about the spectrum of available treatment, and vi) how currently illegitimate service providers can be identified and bogus treatment eliminated.

## Conclusions

This study has highlighted the outpatient nature of available alcohol treatment facilities in Khayelitsha, together with the potential limitations of the available treatment provision, thereby identifying areas where the extant system can be adjusted to improve both service provision and service delivery to facilitate treatment seeking, access and efficacy. The study confirmed that there was limited reach of locally-available self-help networks such as AA and NA for problem drinking males in the Khayelitsha area. Although no respondents reported cultural factors as playing a role, further research with users of services and community members might be needed to examine the impact of social stigma and cultural factors as barriers to treatment seeking for problem drinking.

## Abbreviations

AA: Alcoholics anonymous
DSD: Department of Social Development
NA: Narcotics anonymous
NGO: Non-governmental organisation
